# Colorectal Cancer Prognosis Following Obesity Surgery in a Population-Based Cohort Study

**DOI:** 10.1007/s11695-016-2431-6

**Published:** 2016-11-07

**Authors:** Wenjing Tao, Peter Konings, Mark A. Hull, Hans-Olov Adami, Fredrik Mattsson, Jesper Lagergren

**Affiliations:** 1 Upper Gastrointestinal Surgery, Department of Molecular medicine and Surgery, Karolinska Institutet, Norra Stationsgatan 67, 171 76 Stockholm, Sweden; 2 Leeds Institute of Biomedical and Clinical Sciences, University of Leeds, St James’s University Hospital, Leeds, UK; 3 Department of Epidemiology, Harvard School of Public Health, Boston, MA USA; 4 Department of Epidemiology and Biostatistics, Karolinska Institutet, Stockholm, Sweden; 5 Division of Cancer Studies, King’s College London, London, UK

**Keywords:** Bariatric surgery, Colorectal neoplasms, Mortality, Survival, Registry

## Abstract

**Background:**

Obesity surgery involves mechanical and physiological changes of the gastrointestinal tract that might promote colorectal cancer progression. Thus, we hypothesised that obesity surgery is associated with poorer prognosis in patients with colorectal cancer.

**Methods:**

This nationwide population-based cohort study included all patients with an obesity diagnosis who subsequently developed colorectal cancer in Sweden from 1980 to 2012. The exposure was obesity surgery, and the main and secondary outcomes were disease-specific mortality and all-cause mortality, respectively. Cox proportional hazard survival models were used to calculate hazard ratios (HRs) with 95% confidence intervals (CIs), adjusted for sex, age, calendar year and education level.

**Results:**

The exposed and unexposed cohort included 131 obesity surgery and 1332 non-obesity surgery patients with colorectal cancer. There was a statistically significant increased rate of colorectal cancer deaths following obesity surgery (disease-specific HR 1.50, 95% CI 1.00–2.19). When analysed separately, the mortality rate was more than threefold increased in rectal cancer patients with prior obesity surgery (disease-specific HR 3.70, 95% CI 2.00–6.90), while no increased mortality rate was found in colon cancer patients (disease-specific HR 1.10, 85% CI 0.67–1.70).

**Conclusion:**

This population-based study among obese individuals found a poorer prognosis in colorectal cancer following obesity surgery, which was primarily driven by the higher mortality rate in rectal cancer.

## Introduction

Obesity is associated with increased incidence and decreased survival in several malignancies, including colorectal cancer [1, 2]. Some studies have also shown an overall decreased risk of obesity-related cancer following obesity surgery [3–6], with the exception of colorectal cancer for which an increased risk has been identified [7, 8]. However, the potential effect of obesity surgery on colorectal cancer prognosis has not been studied previously. As obesity plays a negative role in overall cancer diagnostics and treatment [9], weight loss through obesity surgery might have a favourable impact on prognosis. Typically, obesity surgery patients also have more encounters with healthcare that might increase the chance of early cancer detection. However, obesity surgery could have a negative impact on colorectal cancer prognosis. Symptoms caused by a colorectal tumour might be confused with symptoms associated with obesity surgery, such as weight loss, altered bowel habits and abdominal pain, which might delay diagnosis. Furthermore, obesity surgery involves major anatomical and physiological changes of the gastrointestinal tract that might promote malignant transformation of the colorectal mucosa [10]. For example, hyperproliferation of the rectal mucosa has been observed among patients undergoing obesity surgery by means of gastric bypass, which could indicate more aggressive tumour growth [11]. The aim of this study was to elucidate the association between obesity surgery and colorectal cancer prognosis, and we hypothesised that obesity surgery worsens the prognosis for patients with colorectal tumours.

## Methods

### Study Design

This was a nationwide, population-based cohort study including all patients with a hospital discharge diagnosis of obesity in the Swedish Patient Registry combined with a diagnosis of colorectal cancer in the Swedish Cancer Registry from 1st of January 1980 to 31st of December 2012. The Swedish Patient Registry holds information on hospital discharges in Sweden, including admission and discharge dates, main- and co-diagnoses and surgical procedures. This register was 85% nationally complete between 1980 and 1986 and has been 100% complete since 1987 [12]. The registration of surgical procedure codes in the Patient Registry is >95% accurate and >98% complete [13]. The Cancer Registry records cancer cases in Sweden since 1958; it has an overall completeness of 98%, and 99% of the tumours are morphologically verified [14]. The registers can be linked by means of the unique 10-digit personal identity numbers assigned to all Swedish residents upon birth or immigration [15]. The study was approved by the Regional Ethical Review Board in Stockholm, Sweden (reference number 2012/210-31/2). No informed consent from participants is required for register-based studies according to Swedish law.

### Study Cohort

Eligible cohort members were identified from diagnosis codes in the registers: *obesity* was identified from the codes 287, 277, 278A and E66 in the International Classification of Disease (ICD) versions 8 to 10 in the Patient Registry, and *colorectal cancer* was detected from the diagnosis codes 153-154 in ICD7 (corresponding to C18-C20 in ICDO3) in the Cancer Registry. Only patients with colorectal cancer as their first cancer and who received this diagnosis after the date of obesity diagnosis or date of obesity surgery were included in the study. The cohort of obese study participants was grouped into those who had undergone obesity surgery (exposed) and those who had not (unexposed). *Obesity surgery* was ascertained from surgical codes in the Patient Register: 4750-4754 before 1997, and JFD00-01, JDF10-11, JDF20-21 and JFD00 from 1997 and onwards according to the NOMESCO Classification of Surgical Procedures.

### Follow-Up

The main study outcome was colorectal cancer-specific survival, measured through disease-specific deaths, and the secondary outcome was overall survival, measured through all-cause deaths. Dates and causes of death were ascertained through record linkage based on the personal identity numbers from the Swedish Causes of Death Registry. Colorectal cancer-specific deaths were identified through the diagnosis codes 153-154 in ICD 8-9 and C18-C20 in ICD 10. The Causes of Death Registry has nationwide coverage since 1961. Causes of death are reported by the physician issuing the death certificate; ≤1% of the cases lack cause of death data, and the registration of death dates is 100% complete [16]. Migration data were retrieved from the Swedish Registry of the Total Population, and study participants that emigrated during the study period were censored. Data on highest achieved education level were retrieved from the Swedish Education Registry, and were collected to adjust for potential confounding by education. The Registry of the Total Population is updated continuously and the Education Register is updated yearly [17].

### Statistical Analysis

Cox proportional hazard models were used to calculate hazard ratio (HR) with 95% confidence intervals (CI) for disease-specific and all-cause mortality, comparing obese cohort members with and without a history of  obesity surgery prior to their colorectal cancer diagnosis. The analyses were performed for colon and rectal cancer patients combined as well as separately. Time to event was defined as the time elapsed from the date of colorectal cancer diagnosis until the first occurrence of any of the following events: death, emigration or end of follow-up (December 31, 2012). In multivariate modelling, HRs were adjusted for the following potential confounders: sex, age at diagnosis (continuous), calendar period (categorised into 1980–1999 and 2000–2012) and education level (categorised into low [≤9 years], medium [10–12 years] and high [>12 years] based on the Swedish schooling system). We included an interaction term between surgery and tumour location (colon or rectum) to assess differences in HR between tumour sites and evaluated significance with the likelihood ratio test. Model assumptions and fit were assessed formally and graphically. A penalized spline for age was introduced in the colon cancer subgroup analysis in order to satisfy the proportional hazard assumption [18]; smoothing parameters were selected based on the Akaike information criterion [19]. In a separate analysis, we modelled obesity surgery as a categorical variable with four possible values (no surgery, gastric bypass, restrictive procedures [gastric banding and vertical banded gastroplasty] and malabsorptive procedures) to evaluate whether the type of obesity procedure affects colorectal cancer prognosis differently.

Because information on education level was missing for 4% of the patients, we used multiple imputations by chained equations under a missing at random assumption for this variable in the multivariate model [20]. Sensitivity analysis with a complete-case analysis under the missing completely at random assumption, and multiple imputations under the missing-not-at-random assumption yielded similar results [21]. All statistical tests were two-sided, and results were considered significant at a 5% significance level. The statistical software R was used for all statistical analyses [22].

## Results

### Study Participants

**Fig. 1:**
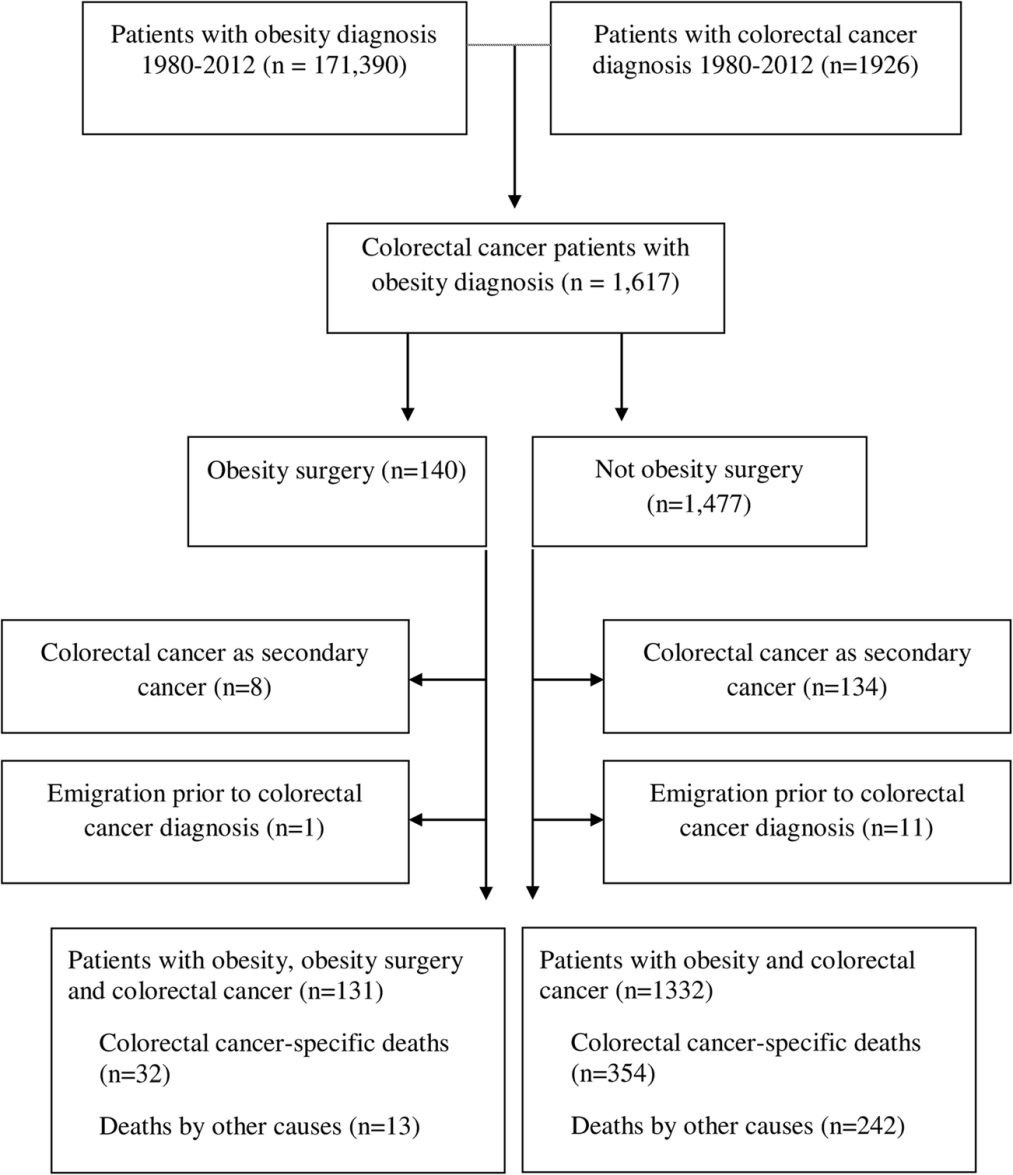
Flowchart for inclusion and exclusion of patients with diagnoses of colorectal cancer and obesity in Sweden from 1980 to 2012, according to obesity surgery status

The study cohort consisted of 1463 patients with an obesity diagnosis followed by a colorectal cancer diagnosis during the study period. Among these patients, 1009 had colon cancer (69%), 449 had rectal cancer (31%) and 5 had both colon and rectal cancer codes. Altogether, 131 (9%) had undergone obesity surgery, while the remaining 1332 patients had not. A flowchart describing the inclusion and exclusion of patients is presented in Fig. 1. Characteristics of the obesity surgery and non-obesity surgery cohort members are presented in Table 1. The obesity surgery group was younger and included more women than the non-obesity surgery group, while diabetes and cardiovascular diseases were more common in non-obesity surgery patients.

**Table 1:** Characteristics of patients with a diagnosis of colorectal cancer and obesity, who had or had not undergone obesity surgery, between 1980 and 2012 in Sweden

	No obesity surgery (*n* = 1332) Number (%)	Obesity surgery (*n* = 131) Number (%)
Age at colorectal cancer diagnosis, years		
<56	240 (18)	62 (47)
≥56	1092 (82)	69 (53)
Sex		
Male	679 (51)	40 (31)
Female	653 (49)	91 (69)
Year of colorectal cancer diagnosis		
1980–1999	413 (31)	30 (23)
2000–2012	919 (69)	101 (77)
Obesity surgery procedure		
Gastric bypass	–	34 (26)^a^
Gastric banding	–	43 (33)^a^
Vertical banded gastroplasty	–	47 (36)^a^
Malabsorptive surgery	–	7 (5)^a^
Education		
Low (≤9 years)	634 (48)	46 (35)
Medium (10–12 years)	460 (35)	61 (47)
High (>12 years)	183 (14)	18 (14)
Missing	55 (4)	6 (5)
Comorbidity		
Diabetes	427 (32)	29 (22)
Cardiovascular disease	289 (22)	13 (10)
Hypertension	481 (36)	39 (30)
Chronic obstructive pulmonary disease	120 (9)	8 (6)
Colorectal cancer	1332^b^	131^b^
Colon cancer	917 (69)	97 (74)
Rectal cancer	419 (31)	35 (26)
Tumour stage (TNM)^c^		
0–I	192 (14)	16 (12)
II–III	304 (23)	32 (24)
IV	107 (8)	19 (15)
Unknown	729 (55)	64 (49)

^a^One patient in the obesity surgery cohort was coded with gastric bypass and non-gastric bypass surgery simultaneously.

^b^One patient in the obesity surgery cohort and four patients in the non-obesity surgery cohort were diagnosed with colon and rectal cancer simultaneously.

^c^Patients for whom data on tumour stage were available. Data on tumour stage were only available in patients diagnosed from 2004 and onward

**Fig. 2:**
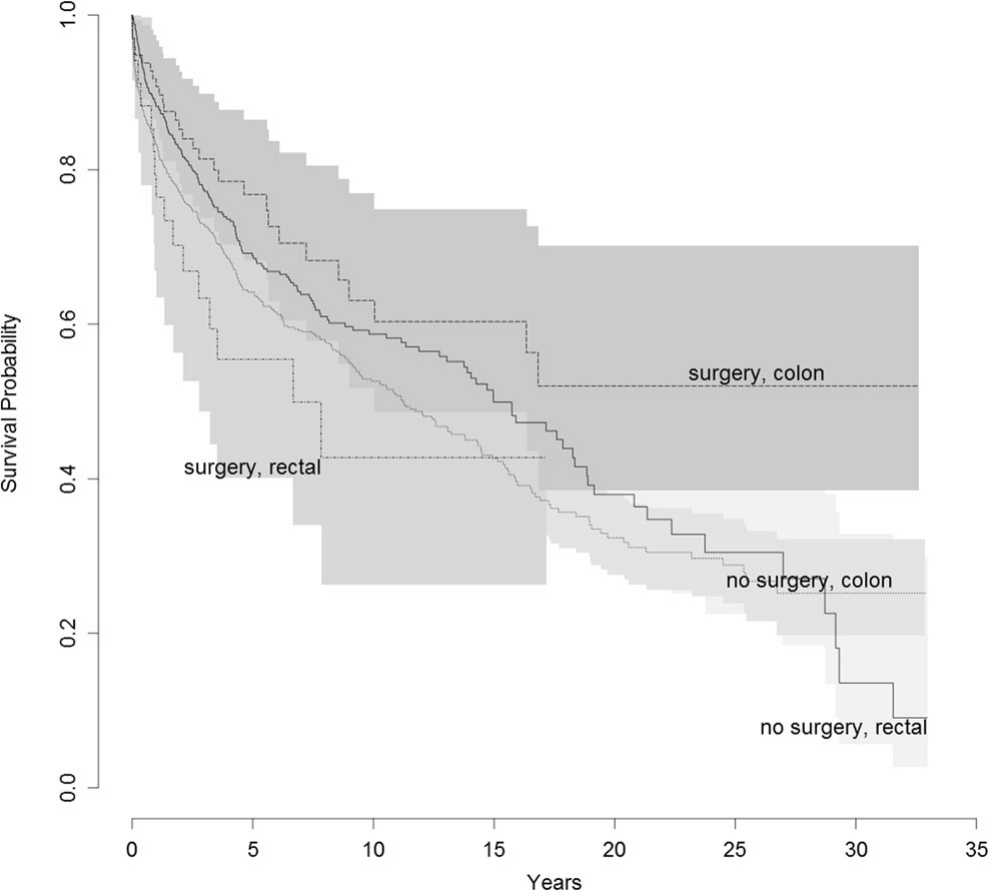
Disease-specific survival proportion of patients with obesity and colon or rectal cancer in Sweden from 1980 to 2012, according to obesity surgery status

Out of 45 (34%) patients in the obesity surgery cohort that died during the study period, 32 (24%) died from colorectal cancer. The number of deaths among the non-obesity surgery cohort members was 596 (45%), of whom 354 (27%) died from colorectal cancer. The median length of follow-up was 3.7 years among obesity surgery and 4.3 years among non-obesity surgery participants. The survival proportions are presented in Fig. 2. Most patients with colorectal cancer as the cause of death died within the first 5 years of diagnosis.

### Colorectal Cancer Survival

Colorectal cancer patients who had undergone prior obesity surgery experienced higher cancer-specific (HR 1.50; 95% CI 1.00–2.19) and overall mortality rates (HR 1.62; 95% CI 1.18 to 2.22) than patients without such surgery (Table 2). Separate analyses of colon and rectal cancer patients revealed no significant difference in mortality rates between obesity surgery and non-obesity surgery patients with respect to colon cancer (disease-specific HR 1.10; 95% CI 0.67–1.70). However, cancer-specific deaths in rectal cancer patients were threefold higher in those who had undergone previous obesity surgery compared to those who had not (disease-specific HR 3.70; 95% CI 2.00–6.90). Overall survival mirrored the disease-specific survival (Table 2). In a separate analysis, obesity surgery was categorized according to type of procedure. The results showed borderline significant association between colorectal cancer-specific mortality and restrictive procedures (HR 1.49; 95% CI 1.00–2.22) and no significant association with gastric bypass or malabsorptive procedures.

**Table 2:** Obesity surgery and all-cause or disease-specific death among obese patients with colorectal cancer, presented as hazard ratio (HR) and 95% confidence interval (CI)

Mortality	No. of patients, surgery	No. of patients, no surgery	Unadjusted HR	95% CI	*P* value	Adjusted HR^a^	95% CI	*P* value
Colorectal cancer								
Disease-specific deaths	32	354	1.09	0.76 to 1.6	0.64	1.50	1.00 to 2.19	0.04
All deaths	45	596	1.30	0.96 to 1.8	0.09	1.62	1.18 to 2.22	<0.001
Colon cancer								
Disease-specific deaths	20^b^	251	0.71	0.45 to 1.10	0.14	1.10	0.67 to 1.70	0.75
All deaths	29	419^b^	0.60	0.41 to 0.87	0.0078	1.28	0.87 to 1.90	0.21
Rectal cancer								
Disease-specific deaths	13^b^	103	1.80	1.00 to 3.30	0.038	3.70	2.00 to 6.90	<0.0001
All deaths	16	177^b^	1.60	0.97 to 2.60	0.064	3.00	1.80 to 5.10	<0.0001

^a^Adjusted for sex, age at diagnosis, year of diagnosis, and education. Missing data on education (4%) was imputed under the missing-at-random assumption

^b^One patient in the obesity surgery cohort was diagnosed with both colon and rectal cancer simultaneously and died of rectal cancer; four patients in the non-obesity surgery cohort were diagnosed with both colon and rectal cancer simultaneously, and two patients died of other causes than colorectal cancer

## Discussion

This study indicates that among obese individuals diagnosed with colorectal cancer, previous obesity surgery had a negative prognostic impact on cancer survival that was primarily driven by the threefold higher rates of cancer deaths in patients with rectal cancer.

Colorectal cancer is the only known malignancy where the risk of being diagnosed with the disease seems to increase after obesity surgery [7, 8]. The present study suggests that this increase translates into poorer survival in rectal cancer but not colon cancer when compared to obese controls. Differences in aetiology and biological mechanisms between colon and rectal cancer might explain the divergent findings between the tumour sites [23]. The somatic mutation profile is similar between colon and rectal tumours, but there are differences in phenotype and effect modifiers [24–26]; it is, e.g. recognised that obesity is a stronger risk factor for colon cancer than rectal cancer [2, 27, 28]. Furthermore, colon and rectal cancers face different treatment regimens. Pre-operative radiotherapy is for example used in rectal cancer but not in colon cancer. We cannot exclude that other confounding factors, e.g. changes in lifestyle factors, may have contributed to the worse prognosis in rectal cancer following bariatric surgery. However, any such confounding should be similar for colon cancer. Moreover, it is likely that lifestyle habits changed to more healthy behaviours and increased health awareness following obesity surgery.

A study of patients undergoing gastric bypass for obesity found hyperproliferation of the rectal mucosa as a result of increased expression of proinflammatory genes from 6 months up to 3 years after surgery [11], indicating potentially more aggressive tumour growth. Our study could, however, not confirm if type of bariatric procedure had an impact on colorectal cancer prognosis. Most of the patients in the cohort had undergone restrictive obesity surgery (69%), and the sample size was not sufficiently powered to evaluate the association between specific types of bariatric procedure and colorectal cancer-specific deaths.

The overall higher rate of deaths observed in obesity surgery patients in this study contradicts previous findings that obesity surgery is protective of mortality. However, our analyses are limited to patients with colorectal cancer, and a majority of these patients succumbed to their cancer (71%). Thus, findings from the all-cause mortality analysis were mainly influenced by disease-specific mortality.

Strengths of this study include the population-based cohort design including all patients with colorectal cancer and obesity diagnosis from nationwide Swedish registers that reduces selection bias and increases generalisability of the results. The high validity of the registers from which data on study exposure and outcomes were obtained decreases the risk of information bias. However, a limitation is that only patients with a recorded diagnosis of obesity were included, which possibly excludes obese patients who are otherwise healthy. The results were adjusted for several confounding factors, but we lacked information on other potential confounders including body mass index. As there have been large changes in treatment options during the long study period, we adjusted for calendar period to account for this variation. Tumour stage is commonly adjusted for in cancer survival studies; however, as obesity surgery might lead to altered pathophysiology, tumour stage could be a mediator in the causal pathway between obesity surgery and colorectal cancer prognosis and were thus not adjusted for in this study. We searched for colorectal cancer deaths in both underlying and contributing causes of death. Whether this inclusion overestimates the number of colorectal cancer deaths is unknown, but there is no reason to believe that any such misclassification would be differential between obesity surgery patients and obese controls. Non-differential misclassification of outcome would only dilute associations, and thus not explain the increased mortality in rectal cancer [29]. Finally, we cannot exclude that the findings in the present study are due to chance, in spite of the strong and statistically significant association between obesity surgery and rectal cancer.

In conclusion, this nationwide, Swedish cohort study with long and complete follow-up found increased mortality from rectal cancer, but not from colon cancer, following obesity surgery. Since this is the first study addressing this question, more research is needed before a definite association can be concluded. If the association is true, clinicians should be made aware of the increased risk and poorer prognosis of rectal cancer in patients with prior obesity surgery.

## References

[1] Bardou M, Barkun AN, Martel M (2013). Obesity and colorectal cancer. Gut.

[2] Campbell PT, Newton CC, Dehal AN (2012). Impact of body mass index on survival after colorectal cancer diagnosis: the cancer prevention study-II nutrition cohort. J Clin Oncol.

[3] Sjostrom L, Gummesson A, Sjostrom CD (2009). Effects of bariatric surgery on cancer incidence in obese patients in Sweden (Swedish obese subjects study): a prospective, controlled intervention trial. Lancet Oncol.

[4] Adams TD, Stroup AM, Gress RE (2009). Cancer incidence and mortality after gastric bypass surgery. Obesity (Silver Spring).

[5] Christou NV, Lieberman M, Sampalis F (2008). Bariatric surgery reduces cancer risk in morbidly obese patients. Surg Obes Relat Dis.

[6] McCawley GM, Ferriss JS, Geffel D (2009). Cancer in obese women: potential protective impact of bariatric surgery. J Am Coll Surg.

[7] Derogar M, Hull MA, Kant P (2013). Increased risk of colorectal cancer after obesity surgery. Ann Surg.

[8] Ostlund MP, Lu Y, Lagergren J (2010). Risk of obesity-related cancer after obesity surgery in a population-based cohort study. Ann Surg.

[9] Tao W, Lagergren J (2013). Clinical management of obese patients with cancer. Nat Rev Clin Oncol.

[10] Quercia I, Dutia R, Kotler DP (2014). Gastrointestinal changes after bariatric surgery. Diabetes Metab.

[11] Kant P, Sainsbury A, Reed KR (2011). Rectal epithelial cell mitosis and expression of macrophage migration inhibitory factor are increased 3 years after Roux-en-Y gastric bypass (RYGB) for morbid obesity: implications for long-term neoplastic risk following RYGB. Gut.

[12] Ludvigsson JF, Andersson E, Ekbom A (2011). External review and validation of the Swedish national inpatient register. BMC Public Health.

[13] Nilsson AC, Spetz CL, Carsjo K (1994). Reliability of the hospital registry. The diagnostic data are better than their reputation. Lakartidningen.

[14] Barlow L, Westergren K, Holmberg L (2009). The completeness of the Swedish Cancer Register: a sample survey for year 1998. Acta Oncol.

[15] Ludvigsson JF, Otterblad-Olausson P, Pettersson BU (2009). The Swedish personal identity number: possibilities and pitfalls in healthcare and medical research. Eur J Epidemiol.

[16] National Board of Health and Welfare (2010). Dödsorsaksstatistik: Historik, produktionsmetoder och tillförliglighet.

[17] Statistics Sweden (2006). Historic Population Register.

[18] Eilers PH, Marx BD (1996). Flexible smoothing with B-splines and penalties. Stat Sci.

[19] Hurvich CM, Simonoff JS, Tsai C (1998). Smoothing parameter selection in nonparametric regression using an improved Akaike information criterion. JRSSB.

[20] Van Buuren S, Groothuis-Oudshoorn K (2011). Mice: multivariate imputation by chained equations in R. J Stat Softw.

[21] Therneau TM, Grambsch PM (2000). Modeling survival data: extending the Cox model.

[22] R: A language and environment for statistical computing. R Foundation for Statistical Computing [computer program]. Version 3.1.2. Vienna: R Core Team; 2015. Available at: http://www.R-project.org/.

[23] Comprehensive molecular characterization of human colon and rectal cancer. Nature. 2012;487(7407):330–7. 10.1038/nature11252 PMC3401966 22810696

[24] Slattery ML, Curtin K, Wolff RK (2009). A comparison of colon and rectal somatic DNA alterations. Dis Colon rectum.

[25] Frattini M, Balestra D, Suardi S (2004). Different genetic features associated with colon and rectal carcinogenesis. Clin Cancer Res.

[26] Einspahr JG, Krouse RS, Yochim JM (2003). Association between cyclooxygenase expression and colorectal adenoma characteristics. Cancer Res.

[27] Larsson SC, Wolk A (2007). Obesity and colon and rectal cancer risk: a meta-analysis of prospective studies. Am J Clin Nutr.

[28] Ma Y, Yang Y, Wang F (2013). Obesity and risk of colorectal cancer: a systematic review of prospective studies. PLoS One.

[29] Rothman KJ, Greenland S, Lash TL (2008). Modern epidemiology.

